# Effect of anti-vascular endothelial growth factor on the surgical outcome of neovascular glaucoma: An overview and meta-analysis

**DOI:** 10.1097/MD.0000000000027326

**Published:** 2021-10-01

**Authors:** Hyung Bin Hwang, Na Young Lee

**Affiliations:** a Department of Ophthalmology, Incheon St. Mary's Hospital, College of Medicine, The Catholic University of Korea, Republic of Korea; b Department of Ophthalmology, Eunpyeong St. Mary's Hospital, College of Medicine, The Catholic University of Korea, Republic of Korea.

**Keywords:** Ahmed glaucoma valve, anti-VEGF, bevacizumab, meta-analysis, neovascular glaucoma

## Abstract

**Background:**

Bevacizumab is known to be very effective in inhibiting ocular neovascularization in neovascular glaucoma (NVG). The purpose of this study is to evaluate the effect of anti-vascular endothelial growth factor on the surgical outcome of Ahmed glaucoma valve implantation (AGVI) in NVG.

**Methods:**

An extensive search of PubMed, EMBASE, and the Cochrane Library was carried out in January 2021 to select relevant studies. The weighted mean difference of the intraocular pressure reduction percentage from baseline to endpoint was used for the primary efficacy estimate. Mantel–Haenszel odds ratios and 95% confidence intervals (CIs) for success rate were employed as secondary efficacy estimates. The number of postoperative interventions and the tolerability estimate for adverse events were also measured using odds ratios. We conducted meta-analyses of fixed effects models using comprehensive meta-analysis software to pool the results of the included studies. Heterogeneity was assessed using Q-value and I^2^ measures.

**Results:**

Nine studies were included in the analysis, encompassing a total of 410 eyes. There was no significant difference in intraocular pressure reduction percentage between the AGVI-only group and the AGVI with adjuvant bevacizumab group (estimate 0.324; 95% CI, −0.278-0.926; *P* = .244). However, the success rate favored the AGVI with adjuvant bevacizumab group (estimate 0.561; 95% CI, 0.097-1.025, *P* = .018).

**Conclusions:**

AGVI with adjuvant bevacizumab had no significant effect on lowering IOP in patients with neovascular glaucoma compared with AGVI alone. However, the final success rate was higher for AGVI with adjuvant bevacizumab treatment than with AGVI alone.

## 1 Introduction

Neovascular glaucoma (NVG) is an aggressive form of secondary glaucoma caused by neovascularization in the iris and the anterior chamber angle. The most common causes of NVG include diabetic retinopathy, central retinal vein occlusion, and carotid ischemic disease.^[1]^ These conditions share a common underlying initiating mechanism as a predisposition for developing NVG retinal ischemia.^[2]^ Treatment of NVG has 2 main components: management of IOP elevation and reduction of the ischemic drive, traditionally through panretinal photocoagulation.^[2,3]^

It is important to treat both elevated intraocular pressure (IOP) and the underlying cause of the disease when managing NVG.^[4]^ Vascular endothelial growth factor (VEGF) is the main causative agent of neovascularization.^[5,6]^ Retinal ischemia has been shown to upregulate VEGF expression, which triggers an angiogenic signaling cascade that promotes neovascularization development in the iris and anterior chamber angle.^[2,5–9]^ Therefore, anti-VEGF treatment is anticipated to play an important role in NVG treatment.

Bevacizumab is an anti-VEGF recombinant humanized monoclonal antibody, and ranibizumab is a recombinant humanized antibody fragment that binds all isoforms of VEGF-A with high affinity. Although the United States Food and Drug Administration (U.S. FDA) has not approved intravitreal injection of bevacizumab (IVB), bevacizumab has been widely used to treat VEGF-mediated ocular conditions, and the outcomes of off-label NVG intravitreal bevacizumab treatments are well known.^[2,10–18]^

NVG is a secondary glaucoma caused by retinal ischemia; therefore, anti-VEGF treatment could potentially influence both the underlying cause of the disease and the secondary elevation in IOP. Moreover, poor surgical success in the treatment of IOP in eyes with NVG suggests the need for an anti-VEGF agent to achieve better outcomes. However, there is no consensus on adjuvant anti-VEGF in eyes with NVG undergoing Ahmed glaucoma valve implantation (AGVI).

In the present study, we conducted a meta-analysis to compare the surgical outcomes of eyes of NVG patients, in terms of the intraocular pressure reduction percentage (IOPR%) and the AGVI success rate between those who received adjuvant bevacizumab with AGVI and those who underwent AGVI alone (without IVB).

## 2 Methods

### 2.1 Search strategy

Searches of PUBMED, EMBASE, and the Cochrane Library databases were conducted, using the terms *Ahmed valve, neovascular glaucoma,* and *bevacizumab*. To identify studies not yet included in the computerized databases, checking of the reference lists of original reports and review articles was carried out manually. The final search was performed in January 2021; we did not restrict the reports and articles based on publication year.

### 2.2 Ethics and dissemination

Ethical approval is not required, because this study is based on existed literature. The findings of this systematic review will be disseminated through a peer-reviewed journal.

### 2.3 Inclusion and exclusion criteria

The inclusion criteria for the published studies were as follows: design, controlled clinical study; population, patients with NVG who underwent AGVI; intervention, IVB injection before AGVI vs AGVI alone; and outcome variables, inclusion of at least 1 of the outcomes of interest discussed below. The exclusion criteria were abstracts from conferences and full texts without raw data available for retrieval, duplicate publications, letters, and reviews. Only the most recent of sequential reports on the same cohort of patients was included. Data that could not be obtained from the most recent publication were obtained from previous reports.

### 2.4 Outcome measures

The IOP reduction percentage (IOPR%) was the primary outcome efficacy measure. The mean value and standard deviation (SD) of the IOPR% were in cases in which authors reported the values directly. For studies that reported only absolute values for the IOP at baseline and at the endpoint, the IOP reduction (IOPR) and the SD of the IOPR (SD_IOPR_) were calculated, as follows: IOPR = IOP_baseline_ − IOP_end-point_, SD_IOPR_ = (SD_baseline_^2^ + SD_endpoint_^2^ − SD_baseline_ × SD_end-pont_)^1/2^. The estimation of IOPR% and the SD of the IOPR% (SD_IOPR%_) were determined as follows: IOPR% = IOPR/IOP_baseline_, SD_IOPR%_ = SD_IOPR_/IOP_baseline_.^[19]^ The success rates, including complete and qualified success rates, were applied. Complete success was defined as achieving the target endpoint IOP without medication, and qualified success was defined as obtaining the target endpoint IOP with or without medication. The proportion of patients who underwent postoperative interventions was designated as the third outcome.

### 2.5 Data extraction

Two investigators independently extracted the data using standardized data abstraction forms. Any disagreements were discussed with a third independent glaucoma specialist. The information collected from these publications included author/s name/s, publication year, study design, study duration, sample size, age, and sex of the study population, IOP measurement, and success rate.

### 2.6 Qualitative assessment

Two authors assessed the quality of the clinical trials that were included in this study using a system reported by Downs and Blacks that assesses both randomized and nonrandomized studies.^[20]^ In the system, 27 items are distributed among 5 subscales regarding reporting (10 items), external validity (3 items), bias (7 items), confounding (6 items), and power (1 item). Discussion with a third investigator was undertaken to reach a consensus when there was any discrepancy in the qualitative assessment. In each trial, the total score was expressed as a percentage of the maximum achievable score. Studies were considered to be of high quality if a quality score was above 50%.

### 2.7 Statistical analysis

Quantitative data were entered into Jamovi software (The jamovi project, 2021, jamovi Version 1.6, Computer Software. Retrieved from https://www.jamovi.org). The pooled odds ratios were calculated for dichotomous outcomes, and the weighted mean difference or standard mean difference was calculated for continuous outcomes; in both cases, 95% confidence intervals (CIs) were reported. A *P* value <.05 was considered to indicate statistical significance for the overall effect. To assess heterogeneity between studies, the I^2^ statistic was calculated. Significant statistical heterogeneity was indicated if *P* < .05 or if the I^2^ measure was above 50%. A fixed-effects model was used to pool results in cases where there was no significant heterogeneity; otherwise, a random-effects model was applied. For the evaluation of the effect of methodological characteristics in terms of study design, a subgroup analysis was performed. The studies were classified as retrospective (Retro), prospective (Pro) nonrandomized, or randomized.

## 3 Results

### 3.1 Overall characteristics of the selected trials and quality assessment

**Figure 1:**
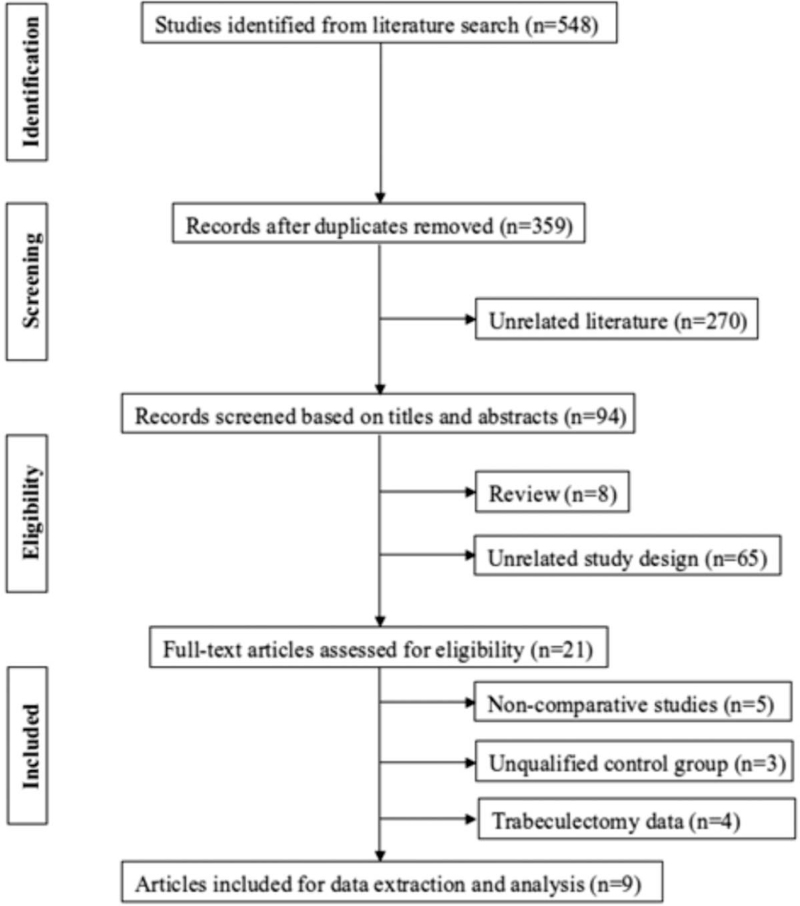
Flow-diagram on identification, screening and inclusion of eligible publications.

From the initially identified articles totalling 359, 350 articles were rejected based on our exclusion criteria. The remaining 9 articles with full text that met the inclusion criteria were assessed and included in this meta-analysis^[21–29]^ (Table 1). A flow diagram of the search results is displayed in Figure 1. In total, 410 eyes were included in the meta-analysis. All studies had a Downs and Blacks score above 50%, fulfilling the quality criteria.

**Table 1:** Characteristics and quality scores of included studies.

						Sex (male/female)		Intervention regimen			
First author (year)	Design	Location	Number of patients	Number of eyes	Mean age	Combination	Surgery only	Surgery	Bevacizumab	Follow-up (mo)	Quality score (%)
Mahdy (2013)	RCT	Egypt	40	40	55/56	12/8	11/9	AGVI	1.25 mg/0.05 ml	18/18	78.1
Eid (2009)	Pro	Saudi Arabia	30	30	56.0/53.7	ns	ns	AGVI	1.25 mg/0.05 ml	12.5/16.4	65.6
Ma (2012)	Retro	Korea	48	52	ns	11/9 (eyes)	16/16 (eyes)	AGVI	1.25 mg/0.05 ml	12/12	62.5
Kang (2013)	Retro	Korea	26	27	54.8/54.3	11/3	11/2	AGVI	1.25 mg/0.05 ml	6/6	50
Sevim (2013)	Retro	Turkey	41	41	65.5/65.8	11/8	13/9	AGVI	1.25 mg/0.05 ml	12/12	68.8
Zhou (2013)	Retro	China	53	53	54.4/57.9	14/11	22/6	AGVI	2.5 mg/0.1 ml	15.1/15.4	65.6
Arcieri (2015)	RCT	Brazil	40	40	59.3/62.4	13/7	11/9	AGVI	1.25 mg/0.05 ml	25.8/28.2	80.2
Olmos (2016)	Retro	USA	151	163	66.1/63.7	27/37	58/41	AGVI	1.25 mg/0.05 ml	12/12	70.3
Kwon (2017)	Retro	Korea	70	70	59.1/57.2	38/7	18/7	AGVI	1.25 mg/0.05 ml	26/27	69.9

AGVI = Ahmed glaucoma valve implantation, Pro = prospective study, RCT = randomized control trial, Retro = retrospective study.

### 3.2 Efficacy analysis

**Figure 2:**
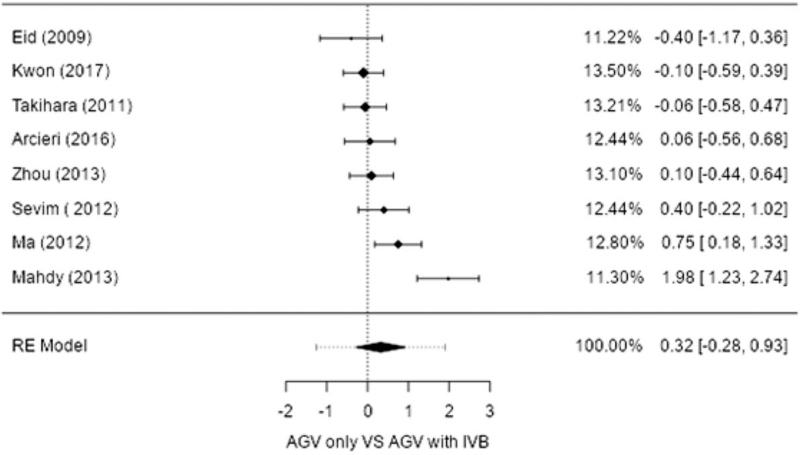
Forest plot of intraocular pressure reduction percentage (IOPR%) between Ahmed glaucoma valve implantation (AGVI) and AGVI with intravitreal injection of bevacizumab (IVB). Error bars represent the 95% confidence intervals of mean. AGV = Ahmed glaucoma valve, RE model = Random effect model.

**IOPR%.** Eight studies compared surgery alone to adjuvant IVB with surgery in terms of the IOPR%. Both procedures showed a significant decrease in IOP, as shown in the combined results. A significant difference in IOPR% was not observed between the 2 groups (estimate 0.324; 95% CI, −0.278–0.926; *P* = .244), with heterogeneity identified (I^2^ = 80.66%; *P* < .001) (Table 2 and Fig. 2).

**Table 2:** Random-effects model and heterogeneity statistics for IOPR (%).

Random-effects model (k = 8)						
	Estimate	se	Z	*P*	CI lower bound	CI upper bound
Intercept	0.324	0.255	1.27	.244	−0.278	0.926

Heterogeneity statistics							
Tau	Tau^2^	I^2^	H^2^	R^2^	df	Q	*P*
0.620	0.384 (SE = 0.2572)	80.66%	5.170	.	7.000	30.026	<.001

Tau^2^ estimator: restricted maximum-likelihood. Knapp and Hartung (2003) adjustment used. CI = confidence interval, IOPR% = intraocular pressure reduction percentage.

#### 3.2.1 Success rate

**Figure 3:**
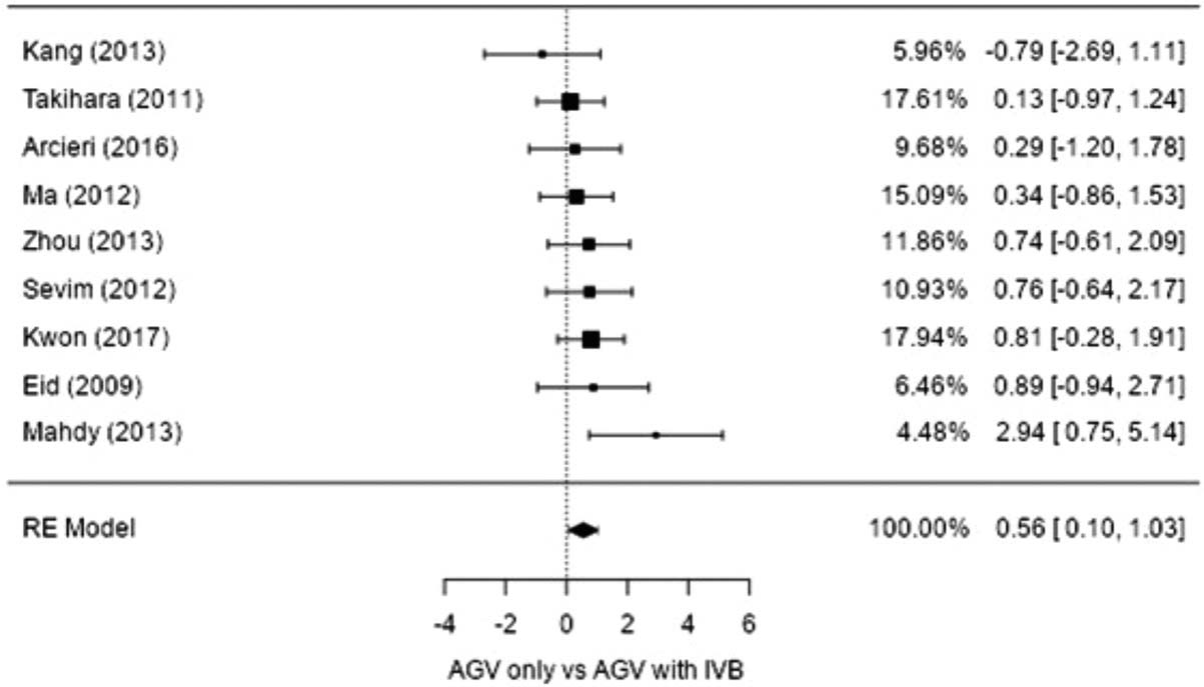
Forest plot of success rate (%) between Ahmed glaucoma valve implantation (AGVI) and AGVI with intravitreal injection of bevacizumab (IVB). Error bars represent the 95% confidence intervals of mean. AGV = Ahmed glaucoma valve, RE model = Random effect model.

The probability of success in all 9 studies was reported, including complete and qualified success. The success rate comparing adjuvant IVB with surgery to surgery alone was in favor of the adjuvant IVB group (estimate 0.561; 95% CI, 0.097-1.025; *P* = .018), with no heterogeneity identified (I^2^ = 0%; *P* = .455) (Table 3 and Fig. 3).

**Table 3:** Random-effects model, heterogeneity statistics and log odds ratio for success rate (%).

Random-effects model (k = 9)						
	Estimate	se	Z	*P*	CI lower bound	CI upper bound
Intercept	0.561	0.237	2.37	.018	0.097	1.025
	.	.	.	.	.	.

Heterogeneity statistics							
Tau	Tau^2^	I^2^	H^2^	R^2^	df	Q	*P*
0.002	0 (SE = 0.2391)	0%	1.000	.	8.000	7.780	.455

Back-transform log odds ratio to odds ratio		
Odds ratio	CI lower bound	CI upper bound
1.752	1.102	2.787

Tau^2^ estimator: restricted maximum-likelihood. CI = confidence interval.

## 4 Discussion

In 1906, Coats described new vessel formation on the iris in eyes with central retinal vein occlusion; this neovascularization of the iris is commonly known as rubeosis iridis.^[30,31]^ Most cases of rubeosis iridis are preceded by retinal ischemia. Diabetic retinopathy, central retinal vein occlusion, and carotid ischemic disease are the most common causes.^[1]^ Although the mechanisms of rubeosis iridis are not fully understood, the following theories have been proposed.^[1,31]^

Retinal ischemia may be 1 factor in the formation of new vessels on the iris and anterior chamber angle, because most of the conditions associated with rubeosis iridis involve reduced perfusion of the retina. Another important mechanism is associated with angiogenesis factors. Four VEGF isoforms (VEGF121, VEGF165, VEGF189, and VEGF206) have been identified, which are generated by alternative mRNA splicing from the same gene.^[31–33]^ VEGF is a potent angiogenic stimulator, promoting several steps of angiogenesis, including proliferation, migration, proteolytic activity, and capillary tube formation, thus playing a crucial role in both normal and pathologic angiogenesis.^[31]^ Vasoinhibitory factors, possibly originating from the vitreous and lens, may cause the ocular tissues to produce substances that inhibit neovascularization,^[34,35]^ which could explain why vitrectomy or lensectomy increases the risk for rubeosis iridis in eyes with diabetic retinopathy.^[31]^

Many studies have attempted to evaluate the value of intraocular anti-VEGF therapy. Some of these reports involved NVG patients with either diabetes or central retinal vein occlusion in whom bevacizumab was injected into the vitreous cavity or in the anterior chamber before or concomitant with panretinal photocoagulation.^[29]^ All treated eyes had significant regression of new anterior segment vessels within 48 hour. The effect of bevacizumab lasted for a number of weeks and, thereafter, new vessel formation was noted to resume in some eyes.^[31]^ Although anti-VEGFs are effective for reducing iris and anterior chamber neovascularization, little is known about their long-term effects on NVG development over time.

Some have also attempted to evaluate the value of intraocular anti-VEGF therapy with bevacizumab as an adjunctive treatment of NVG.^[36]^ Intraocular anti-VEGF therapy showed significant regression of new vessels in the anterior segment within 48 hour; however, the effect of bevacizumab did not last for a long time in all eyes. IVB serves as an effective temporizing treatment, but is not a replacement for close monitoring and definitive NVG treatment. Although IVB is a very simple and effective procedure the possibility of adverse effects should always be kept in mind. Since IVB often causes IOP elevation, and rarely can cause hyphema and choroidal hemorrhage, special attention is required in diseases with neovascularization and uncontrolled IOP such as NVG. In very rare cases, IVB is known to be associated with serious complications such as rhegmatogenous retinal detachment, macular infarction, and ocular ischemic syndrome.^[37]^

In this study, 9 articles were reviewed covering 410 eyes from 340 patients. The combined treatment with adjuvant IVB showed better IOP lowering efficacy, comparable with that of AGVI alone; however, there was no significant IOPR% reduction from baseline (estimate 0.324; 95% CI, −0.278-0.926; *P* = .244). The combined treatment was more likely to achieve surgical success (estimate 0.561; 95% CI, 0.097-1.025, *P* = .018). The data included in this study were pooled from trials of different durations, ranging from 6 to 28 months. We had to compromise by choosing endpoint data, due to the lack of data documented in all phases of the follow-up and trials with different durations. Although the underlying mechanisms of adjuvant IVB to AGVI are unclear, the possible reasons are as follows. First, adjuvant IVB itself has an effect on the underlying disease process responsible for NVG. Second, the bevacizumab-mediated reduction in VEGF concentrations in the anterior and posterior chambers may contribute to a reduction in neovascularization and synechiae of the angle and an increase in anti-fibrotic activity. This would result in a decrease in the wound healing process and inflammatory reaction, which would promote greater success regarding the AGVI procedure.

Previously, we reported that the combined treatment with adjuvant IVB is associated with a significantly lower frequency of hyphema compared with filtering surgery alone (odds ratio  = 0.148; 95% CI, 0.081-0.269; *P* = .000) in NVG patients. Moreover, adjuvant IVB injection has been linked to better outcomes after filtering surgery in patients with NVG. Taken together with the results from the current study, adjuvant IVB could be considered as a safe and effective option to AVGI in patients with NVG.

Our study had several limitations. First, publication bias could not be fully excluded. Despite the fact that we performed both electronic and manual searches to identify all potentially relevant studies, our results must be interpreted carefully. Second, measurement bias may have resulted from the use of masking in 1 of the 9 studies. Third, among the 9 studies, 3 were RCTs, 5 were retrospective, and 1 was nonrandomized and prospective. Sufficient information with regard to how the RCT was implemented and a description of the implementation of allocation concealment were provided in only the RCT study; this may have resulted in selection bias.

Despite these limitations, our meta-analysis results provide a new perspective regarding NVG treatment. This is the first meta-analysis to evaluate specifically whether adjuvant IVB with AGV implantation has a higher success rate than AGVI alone in patients with NVG. Although, AGVI with adjuvant bevacizumab showed no significant IOPR% reduction in patients with NVG, compared to AGVI alone, the present meta-analysis showed that the final success rate was higher for AGVI with adjuvant bevacizumab than AGVI alone. In conclusion, regarding the poor surgical success in eyes with NVG, adjuvant bevacizumab is recommended to improve the surgical success rate of AGVI in NVG patients.

## Author contributions

**Conceptualization:** Hyung Bin Hwang and Na Young Lee.

**Data curation:** Hyung Bin Hwang and Na Young Lee.

**Formal analysis:** Hyung Bin Hwang and Na Young Lee.

**Investigation:** Hyung Bin Hwang and Na Young Lee.

**Supervision:** Na Young Lee.

**Validation:** Hyung Bin Hwang and Na Young Lee.

**Visualization:** Hyung Bin Hwang and Na Young Lee.

**Writing – original draft:** Hyung Bin Hwang and Na Young Lee.
